# Direct Telephonic Communication in a Heart Failure Transitional Care Program: An observational study

**DOI:** 10.4021/cr296e

**Published:** 2013-10-15

**Authors:** Ken S. Ota, David S. Beutler, Hassam Sheikh, Jessica L. Weiss, Dallin Parkinson, Peter Nguyen, Richard D. Gerkin, Akil I. Loli

**Affiliations:** a Department of Transitional Care Medicine, Banner Good Samaritan Medical Center, Phoenix, Arizona, USA; b Cardiology Fellowship Program, Banner Good Samaritan Medical Center, Phoenix, Arizona; c Internal Medicine Residency Program, Banner Good Samaritan Medical Center, Phoenix, Arizona, USA

**Keywords:** Transitional care, Transitionalist, Heart failure, Telephone, Telemonitoring

## Abstract

**Background:**

This study investigated the trend of phone calls in the Banner Good Samaritan Medical Center (BGSMC) Heart Failure Transitional Care Program (HFTCP). The primary goal of the HFTCP is to reduce 30-Day readmissions for heart failure patients by using a multi-pronged approach.

**Methods:**

This study included 104 patients in the HFTCP discharged over a 51-week period who had around-the-clock telephone access to the Transitionalist. Cellular phone records were reviewed. This study evaluated the length and timing of calls.

**Results:**

A total of 4398 telephone calls were recorded of which 39% were inbound and 61% were outbound. This averaged to 86 calls per week. During the “Weekday Daytime” period, Eighty-five percent of the totals calls were made. There were 229 calls during the “Weekday Nights” period with 1.5 inbound calls per week. The “Total Weekend” calls were 10.2% of the total calls which equated to a weekly average of 8.8.

**Conclusions:**

Our experience is that direct, physician-patient telephone contact is feasible with a panel of around 100 HF patients for one provider. If the proper financial reimbursements are provided, physicians may be apt to participate in similar transitional care programs. Likewise, third party payers will benefit from the reduction in unnecessary emergency room visits and hospitalizations.

## Introduction

Heart failure (HF) is a complex, chronic health condition affecting 5.1 million people in the United States (U.S.) [1]. The prevalence of HF is accelerating by a tremendous rate with a projected increase of 25% by the year 2030. In 2009, the number of HF-related deaths was 274,601, with HF being the primary diagnosis in 56,410 of those cases. Approximately 50% of patients diagnosed with HF will die within 5 years of their initial diagnosis.

Frequent and expensive hospitalizations cause HF to be an overwhelming burden on the U.S. healthcare system. In 2010, there were 1.23 million hospital discharges in which HF was the primary reason for hospitalization [1]. Among those 65 years and older, HF is the most common reason for hospitalization with a 27% re-hospitalization rate within 30 days [2]. The American Heart Association (AHA) estimated that in 2013 the total cost of HF would be $32 billion and that number is projected to increase to $70 billion by 2030 [1].

The Centers for Medicaid and Medicare Services (CMS) have begun imposing reimbursement penalties to hospitals with greater-than-expected readmission rates [3, 4]; thus physicians and hospital administrators need to implement innovative care practices to prevent these financial losses. The 2009 Focused Update of the American College of Cardiology (ACC)/AHA Guidelines for the Diagnosis and Management of Heart Failure in Adults established a new Class 1B recommendation that states “Post-discharge systems of care, if available, should be used to facilitate the transition to effective outpatient care for patients hospitalized with heart failure” [5].

At Banner Good Samaritan Medical Center (BGSMC), we are addressing the issue of frequent heart failure readmissions through the creation of the physician-directed Heart Failure Transitional Care Program (HFTCP). This pilot program focuses specifically on improving the post-hospitalization care transition process for patients admitted for acute decompensated heart failure (ADHF) as a primary or secondary diagnosis. The goal of the HFTCP is to increase quality of care by providing personalized, aggressive transitional care during the post-hospitalization period in order to reduce the number of emergency room visits and hospital readmissions. The HFTCP is a multipronged approach that utilizes around-the-clock patient-provider telephone access, patient specific heart failure education, home and skilled nursing facility visits, and an outpatient intravenous (IV) diuresis center to provide a fluid transfer between the inpatient setting and the community [6].

One of the key elements of the HFTCP is providing patients with direct patient-physician telephone access. This type of access which allows patients to communicate with their physician also provides additional opportunities for the physician to educate patients about important lifestyle changes, such as dietary modifications, and allows the physician to teach patients to recognize the signs and symptoms of impending ADHF.

To date, there are no published studies that specifically address the time commitment required to provide HF patients with direct patient-provider telephone access. The aim of this study is to investigate the trend of the timing and volume of telephone calls in the BGSMC HFTCP to better understand patient needs and provider exigencies.

## Subjects and Methods

### Setting

This study was conducted at BGSMC, which is one of the 10 Banner Health hospitals in Arizona. BGSMC is a 668-bed tertiary care hospital located in downtown Phoenix, Arizona. In 2011, there were 43,777 inpatient visits and 64,913 emergency room (ER) visits with approximately 650 HF admissions. BGSMC serves a large number of indigent individuals in the Phoenix metropolitan area. The hospital has eight residency programs and nine fellowship programs including cardiology.

### Population

The study population included 104 HF patients admitted to BGSMC that were subsequently enrolled in the HFTCP from September 2, 2011 to August 23, 2012.

### Screening and enrollment

BGSMC cardiologists and hospitalists were educated on the goals of the HFTCP. Program participants were obtained by referrals from hospital physicians discharging HF patients from the inpatient setting. The HF Transitionalist met with patients prior to discharge to discuss the goals of the HFTCP and screen patients for eligibility into the program. The HFTCP included any patient with ADHF from heart failure with preserved ejection fraction or heart failure with reduced ejection fraction, either as a primary or secondary diagnosis, and who lived within a 15-mile radius of BGSMC. Patients with end stage renal disease requiring dialysis and patients with active illicit drug use were excluded from the program. This study was approved by the BGSMC Institutional Review Board, and researchers adhered to Health Insurance Portability and Accountability Act (HIPAA) regulations.

### Intervention

The Transitionalist who ran the HFTCTP during this study period was a single physician Board Certified in Family Medicine with special heart failure training. Prior to the patients’ discharge from the hospital, the Transitionalist discussed the goals of the program with the patients. Patients were provided with direct around-the-clock access to the Transitionalist via his cell phone. Patients were encouraged to notify him of any new symptom concerning for HF decompensation, or to notify him if they had other non-HF related symptoms that would prompt them to go to the emergency department. After hospital discharge, the Transitionalist met with all of the patients in their homes, his office, or a skilled nursing facility within 72 hours of discharge. The Transitionalist called the patients one to five times per week during the first 30 days post-discharge based on each individual patient’s need. In addition, the Transitionalist helped manage selected patients in the hospital’s outpatient observation unit or outpatient infusion room for IV diuretic therapy in order to provide aggressive treatment for early volume overload without requiring an official hospital admission [6].

### Data

The telephone records from the Transitionalist’s dedicated cellular phone (Verizon Wireless) provided by BGSMC were imputed onto Microsoft® Office Excel 2010 spreadsheets. Telephone calls were categorized by date, day of the week, hour of the day, call length, inbound (patient initiated), and outbound (Transitionalist initiated). Phone conversations included calls from patients, calls to patients, and calls to and from their respective outpatient physicians, caregivers, skilled nursing facilities, and pharmacies.

Calls were assigned to the following categories: “Weekday Daytime” (07:00 to 18:59, Monday through Friday), “Weekday Extended Hours” (07:00 to 21:59 Monday through Thursday and 7:00 to 18:59 on Friday), “Weekday Nights” (19:00 to 06:59, Monday through Thursday), “Total Weekend” (19:00 Friday through 6:59 Monday), “Weekend Daytime” (7:00 to 18:59 Saturday and Sunday), and “Weekend Nights” (19:00 to 06:59, Friday, Saturday and Sunday).

Descriptive statistics were used to report calls by number, hour of the day and by day of the week. Values were reported as means and standard deviations. Percentage of calls was reported to further categorize calls by day and hour. Interquartile range was used to describe the duration of the calls.

## Results

Over the course of 51 weeks, 104 HF patients were enrolled in the HFTCP. The demographics of the patients in the HFTCP are reported in an antecedent article authored by the principle investigators of this study [7]. In brief, the patients’ average age and NYHA class were 67 and 2.8, respectively, and all patients enrolled in the HFTCP were deemed to be high-risk for readmission by at least one of their inpatient physicians (which prompted the referral in the first place). The original article published by the authors of this study showed a pre-enrollment 30-day readmission rate for ADHF of 26.0% and a post-enrollment rate of 4.1% (P < 0.001), and the all-cause pre-enrollment 30-day readmission rate was 28.8% with a post-enrollment rate of 8.2% (P = 0.002) [7].

A total of 4398 telephone calls were recorded. Of these calls, 1719 (39%) were inbound and 2679 (61%) were outbound. On average, the Transitionalist handled 86 calls per week.

The number of calls in each of the different time periods is shown in Table 1. In summary, during the “Weekday Daytime” period (Monday through Friday 7:00 to 18:59), 3721 (85 % of the 4398 totals calls) were made or received, and of the 3950 total week day calls, 3863 (98%) calls were made or received between 7:00 and 21:59, the “Weekday Extended Hours” period. During the “Weekday Nights” period, (Monday through Thursday 19:00 to 6:59), there were 229 calls which made up 5% of the total calls and 5.8% of the 3950 total weekday calls. This averaged 4.5 total evening calls per week, but since only 79 of the total “Weekday Night” calls were inbound, this averaged to 1.5 inbound “Weekend Night” calls per week. The total number of “Weekend” (19:00 Friday evening through 6:59 Monday morning) calls was 448 (10.2% of the total calls) which equates to an average of 8.8 weekend calls of which 2.9 were inbound. Of the weekend calls, 115 (26.2%) were between 19:00 and 6:59 which averaged to 2.25 night time weekend calls with only 0.9 inbound “Weekend Night” calls.

**Table 1:** Distribution of Phone Calls Among Various Time Periods

Time period	Total number of calls			Percent of total calls		
	In	Out	Total	In	Out	Total
Weekday total calls	1572	2378	3950	36%	54%	90%
Weekday daytime	1493	2228	3721	34%	51%	85%
Weekday extended hours	1544	2319	3863	35%	53%	88%
Weekday nights	79	150	229	2%	3%	5%
Weekend total calls	147	301	448	3.3%	6.8%	10.2%
Weekend daytime	100	233	333	2.3%	5.3%	7.6%
Weekend nights	47	68	115	1.1%	1.5%	2.6%
Total	1719	2679	4398	39%	61%	

Calls were placed into the following categories: “Weekday Daytime” (7:00 to 18:59, Monday through Friday), “Weekday Extended Hours” (07:00 to 21:59 Monday through Thursday and 7:00 to 18:59 on Friday), “Weekday Nights” (19:00 to 06:59, Monday through Thursday), “Weekends” (19:00 Friday through 6:59 Monday), “Weekend Daytime” (7:00 to 18:59 Saturday and Sunday), and “Weekend Nights” (19:00 to 06:59, Friday, Saturday and Sunday).

**Figure 1:**
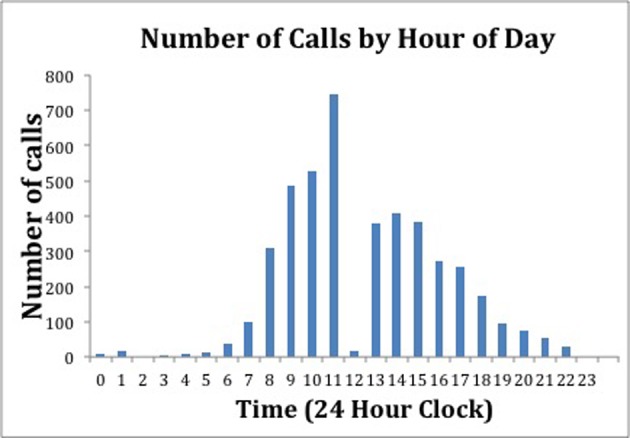
This figure shows the timing of calls by hour of the day over the course of the 51 week study period.

**Figure 2:**
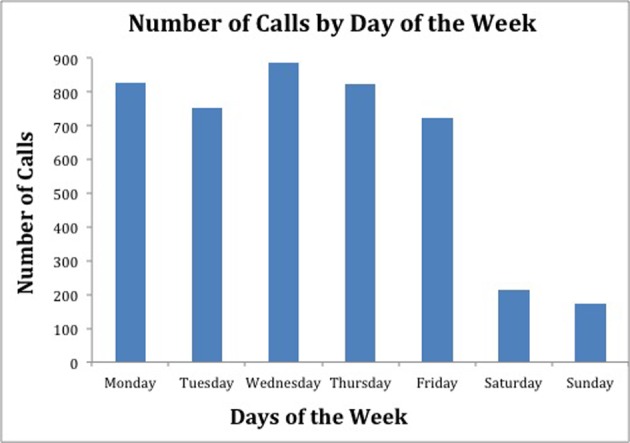
This figure shows the distribution of calls over the days of the week for the 51 week study period.

Figure 1 illustrates the number of calls by hour of the day over the course of the 51 week study period. Interestingly, few calls were made or received during the noon hour compared to the rest of the “Daytime” hours. Figure 2 shows the distribution of the calls made on each day of the week. There were a similar number of calls made on each of the days of the work week (Monday through Friday). The number of calls on Saturdays and Sundays were also similar to each other.

**Figure 3:**
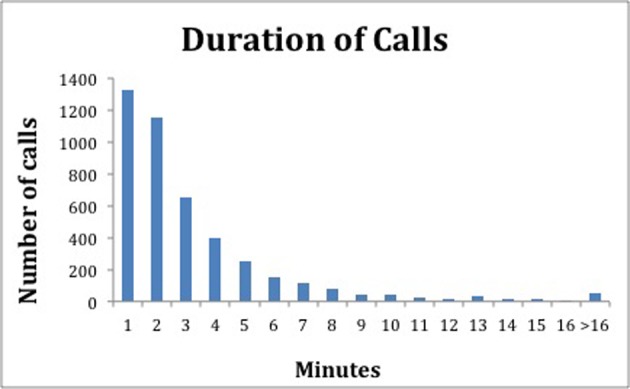
This figure shows the distribution of all phone calls made by duration over the 51 week study period.

The average duration of all calls was 3.6 minutes (SD 3.8), with the inbound calls averaging 3.4 minutes (SD 3.7) and outgoing calls averaging 3.2 minutes (SD 3.3). It appears that the duration of calls among the different time periods was similar and did not vary if the calls were inbound or outbound. Figure 3 shows the distribution of the duration of all calls. The interquartile range of all the calls was between 1 to 4 minutes.

## Discussion

At BGSMC, the physician-directed HFTCP aims to improve the post-hospitalization care transition process for patients admitted for ADHF. Results from the initial study of the HFTCP have shown this program to be effective in significantly reducing all-cause 30-day re-hospitalizations [7]. One of the key components of this program is direct 24-hour daily patient-provider telephone communication. Although many providers may have reservations about the potential time commitment to provide such access, our general experience is that direct provider-patient telephone contact is indeed feasible even in a population that consists of chronically ill and elderly HF patients with multiple comorbidities.

The majority of phone calls in this study were during the “Weekday Daytime” period (84.6%) and less than half the calls were inbound (39%) (Table 1). Inbound after-hours calls comprised only a small percentage of the total phone calls managed by the Transitionalist; there was a weekly average of 1.5 inbound night weekday and 0.9 inbound night weekend calls. This study shows that providing HF patients in a transitional care program with direct access to their physician via cell phone does not appear to be overwhelming. Addressing certain follow-up care issues by phone may, in fact, save time for physicians because fewer of their patients will be significantly symptomatic when they present to the office for follow up appointments. Ideally direct patient access to physicians will also decrease the number of repeat HF emergency room visits [2].

This study shows that this type of program with around-the-clock telephone access for patients is feasible from the physician standpoint. In order to entice physicians to make the sacrifice to take around-the-clock telephone calls from patients, Medicare and other third party payers should explore reimbursing physicians for taking these calls. By preventing unnecessary emergency room visits and inpatient hospital stays, we believe that third party payers will save money with transitional care programs like the BGSMC HFTCP. Full cost analysis will need to be performed to definitively prove this. Currently Medicare provides a bundle payment for transitional care services of about $200 [8]. We believe that the 24-hour telephone access for patients to physicians in a transitional care program should be reimbursed at a higher bundle payment or on a per patient inbound call basis for such access [9].

The benefit of providing patients with a direct line of communication to their physician has previously been demonstrated [10]. An Israeli study assessing patients’ preferences for the various means of communication found that patients generally prefer to contact their physicians by telephone instead of e-mail. These same authors published another study revealing that physicians also favor providing their cell phone numbers rather than their e-mail addresses to patients; although 65% of physicians interviewed in the above mentioned Israeli study felt that the availability of cell phone numbers to patients would infringe upon their privacy after formal work hours [11]. Understandably, many physicians will be hesitant to incorporate direct telephone access into their care practices because of the fear of overextending their workload and hours.

Structured post-discharge follow up protocols have been shown to help prevent and reduce HF hospitalizations, especially 30-day readmissions. Inglis et al. [12] examined several different HF monitoring programs. They concluded that structured telephone contact dramatically reduced HF hospitalizations and healthcare costs, and improved patients’ quality of life. DeWalt and colleagues [13] reported a 44% reduction in the number of hospitalizations and mortalities when patients were educated about how to monitor and control their HF symptoms. They stressed the importance of follow-up phone calls to aid in the care of HF patients. Surprisingly, a study from Northwestern University on telephone counseling for HF patients found that despite the fact that most HF patients were aware of the dangers of a high-salt diet, 24% and 16% were not aware that shortness of breath and leg or ankle swelling, respectively, were signs of ADHF [14]. Phone calls that educate patients on the significance of early clinical symptom detection of ADHF may be crucial for the prevention of re-hospitalizations.

We believe that the approach used in the HFTCP is superior to a nurse-supervised tele-monitoring program in triaging and treating patients when there is a question of imminent ADHF. A 2010 study in the New England Journal of Medicine showed that telemonitoring for HF patients does not necessarily improve patient outcomes [15]. Casas JP et al. [16] showed that data obtained via tele-monitoring can provide a false sense of assurance to both the provider and the patient because indicators of adverse events may be missed in the absence of direct provider-patient interaction. We believe that the synergy of direct telephonic communication between provider and patient and face-to-face interaction is the key to optimal care in the HFTCP.

As the HFTCP at BGSMC continues to grow, we will have a better understanding of the ideal provider-to-patient ratio required to efficiently coordinate a larger transitional care program. Currently our results show that the telephone call burden can be managed by an on-call provider.

### Limitations

It is unclear how many unnecessary after-hours calls occurred and could have waited until normal business hours the next day. Financial analysis of this program has yet to be fully performed. We recognize that as the volume of patients enrolled in the HFTCP increases, the demand on the Transitionalist will increase as well. To address this concern after the study period was completed, the HFTCP has added an advanced care provider to aid with after-hours telephone calls.

### Conclusion

A key component of the BGSMC HFTCP is direct around-the-clock patient-provider telephonic communication that allows for quick assessment and coordination of care with the goal of decreasing emergency room visits and avoidable hospitalizations. This program reduces unnecessary hospitalizations and reduces costs while giving patients the security of having immediate access to their provider. Although many physicians may have reservations about the potential time commitment to such access, our general experience is that direct, around-the-clock physician-patient telephone contact is indeed feasible for the on-call physician. If the proper financial reimbursements are provided, physicians may be apt to participate in similar transitional care programs. Likewise, third party payers will benefit from the reduction in unnecessary emergency room visits and hospitalizations.
